# Harnessing NK Cells for Cancer Treatment

**DOI:** 10.3389/fimmu.2019.02836

**Published:** 2019-12-06

**Authors:** Paola Minetto, Fabio Guolo, Silvia Pesce, Marco Greppi, Valentina Obino, Elisa Ferretti, Simona Sivori, Carlo Genova, Roberto Massimo Lemoli, Emanuela Marcenaro

**Affiliations:** ^1^Clinic of Hematology, Department of Internal Medicine (DiMI), University of Genoa, Genova, Italy; ^2^Ospedale Policlinico San Martino IRCCS, Genova, Italy; ^3^Department of Experimental Medicine, University of Genoa, Genova, Italy; ^4^Centre of Excellence for Biomedical Research, University of Genoa, Genova, Italy; ^5^Lung Cancer Unit, Ospedale Policlinico San Martino IRCCS, Genova, Italy

**Keywords:** NK cells, NK cell receptors, immune checkpoint blockade, immunotherapy, solid tumors, hematological malignancies, adoptive NK cell therapy

## Abstract

In the last years, natural killer (NK) cell-based immunotherapy has emerged as a promising therapeutic approach for solid tumors and hematological malignancies. NK cells are innate lymphocytes with an array of functional competences, including anti-cancer, anti-viral, and anti-graft-vs.-host disease potential. The intriguing idea of harnessing such potent innate immune system effectors for cancer treatment led to the development of clinical trials based on the adoptive therapy of NK cells or on the use of monoclonal antibodies targeting the main NK cell immune checkpoints. Indeed, checkpoint immunotherapy that targets inhibitory receptors of T cells, reversing their functional blocking, marked a breakthrough in anticancer therapy, opening new approaches for cancer immunotherapy and resulted in extensive research on immune checkpoints. However, the clinical efficacy of T cell-based immunotherapy presents a series of limitations, including the inability of T cells to recognize and kill HLA-I^neg^ tumor cells. For these reasons, new strategies for cancer immunotherapy are now focusing on NK cells. Blockade with NK cell checkpoint inhibitors that reverse their functional block may overcome the limitations of T cell-based immunotherapy, mainly against HLA-I^neg^ tumor targets. Here, we discuss recent anti-tumor approaches based on mAb-mediated blocking of immune checkpoints (either restricted to NK cells or shared with T cells), used either as a single agent or in combination with other compounds, that have demonstrated promising clinical responses in both solid tumors and hematological malignancies.

## NK Cell: an “Efficient” Tool for Immunotherapy

Immunotherapy is an innovative approach for the treatment of cancer and is based on the idea of harnessing the immune system to target tumors. Recently, immunotherapy, and in particular immune checkpoint (IC) blockade therapy, has represented a significant step forward for cancer treatment (1–5). Two inhibitory ICs, CTLA-4 (6) and PD-1 (7), received great attention, since the inhibition of CTLA-4 or PD-1 signaling significantly improved the survival of patients with metastatic solid cancers. Given the clinical efficacy of PD-1 and/or CTLA-4 blockade in patients with untreatable solid and hematological cancers (1–5, 8), much attention has been given to IC receptors and their cognate ligands.

Currently, one of the major challenges in immune-oncology is the understanding of the mechanisms of IC inhibitor resistance (indeed, only a fraction of patients respond to immunotherapy), to increase the proportion of patients benefitting from such treatment, and to control treatment toxicity.

A critical point is that the clinical effect of the PD-1/PD-Ls blockade has been conventionally attributed to the restoration of cytotoxic lymphocyte activity. However, a partial or complete loss of HLA-I expression is one of the most frequent mechanisms of tumor escape from T-cell control in different human tumor types (9). In this scenario, the role of the “innate counterpart” of cytotoxic T cells, the natural killer (NK) cells, which show the ability to recognize and kill tumor cells regardless of HLA-I expression, appears to be crucial (10–12).

NK cells were first identified in the mid-1970s as a unique lymphocyte subset able to detect and rapidly kill abnormal cells without prior sensitization or recognition of specific tumor antigens, thus preventing the development of many cancers (13, 14). In the late 1980s, the observation that NK cells could kill a lymphoma cell line that had lost MHC class I surface molecules, while the original MHC class I+ cells were resistant to lysis, led to the formulation of the “missing self-hypothesis” that stated that NK cells are able to sense the absence of “self” MHC class-I molecules on target cells (15, 16). In the 1990s, this hypothesis was confirmed by the discovery of inhibitory (17, 18) and activating NK receptors (19). In humans, the main inhibitory receptors are represented by the inhibitory killer Ig-like receptors (KIRs), recognizing allotypic determinants shared by groups of HLA class-I alleles (20, 21) and by the CD94/NKG2A heterodimer (22), specific for the non-classical HLA-E molecule.

Inhibitory KIRs are type I molecules with two (KIR2D) or three (KIR3D) highly polymorphic extracellular Ig-like domains followed by long (L) cytoplasmic tails harboring two ITIMs, able to transduce an inhibitory signal through the recruitment of tyrosine phosphatases. The four main inhibitory KIRs are specific for epitopes shared by distinct groups of HLA class I allotypes. In particular, KIR2DL1 recognizes HLA-C2 epitope, while KIR2DL2/L3 recognizes HLA-C1 epitope. KIR3DL1 is specific for HLA-B or HLA-A molecules sharing the Bw4 public epitope (Bw4I80 or Bw4T80), and KIR3DL2 binds HLA-A^*^03 and -A^*^11 allotypes (18, 21).

The activating NK cell receptors include a series of non-HLA-specific receptors and co-receptors able to induce NK cell triggering by directly interacting with ligands overexpressed or expressed *de novo* on tumor-transformed or virus-infected cells (23–25).

**Figure 1:**
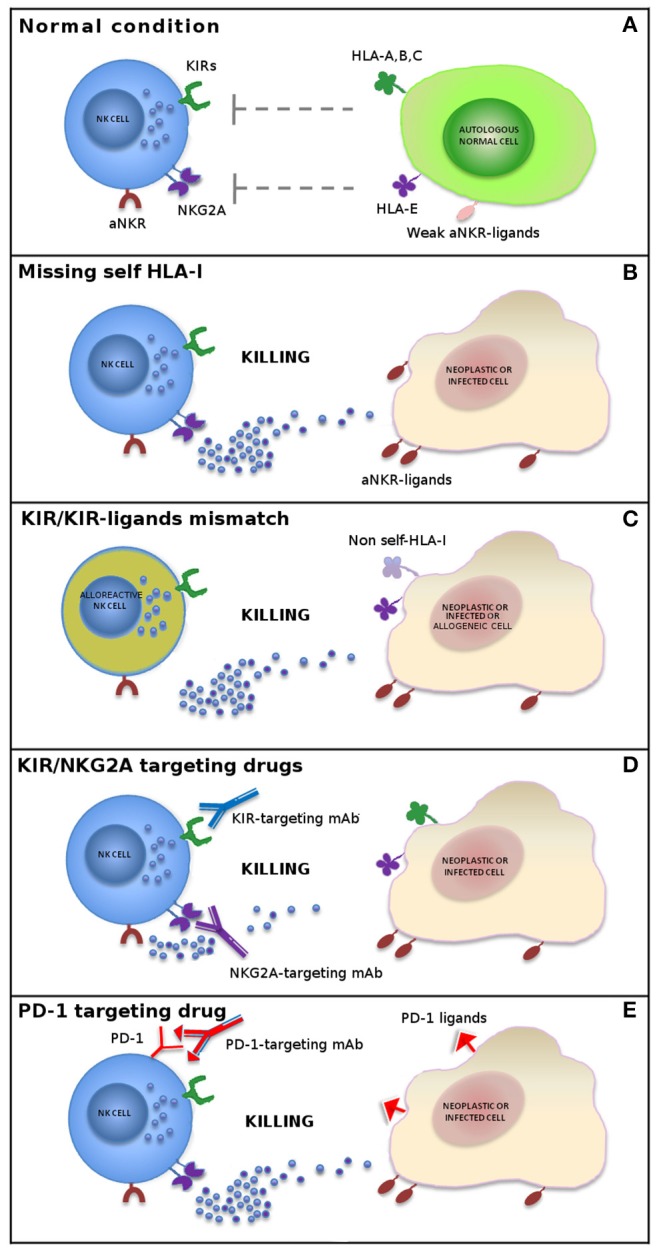
Mechanisms of NK cell-mediated killing. In physiological conditions, NK cell activity is tightly regulated by a complex interplay between inhibitory and activating receptors that prevents killing of normal autologous cells expressing an appropriate level of all self-HLA alleles and low/negative levels of ligands for non-HLA-specific activating receptors (aNKR) **(A)**. Downregulation of HLA-I molecules on neoplastic or infected cells induces NK-mediated killing by a “missing-self” recognition mechanism. NK cell activating receptors are co-responsible in inducing NK cell triggering by interacting with ligands (aNKR-ligands) overexpressed or expressed *de novo* on tumor-transformed or virus-infected cells **(B)**. Allogeneic (alloreactive) donor NK cells are able to kill neoplastic cells of the recipient expressing non-self allotypic determinants on HLA-I molecules (“KIR/KIR-ligand mismatch”) and to control infections with a limited risk of toxicity (e.g., GvHD and HvG) **(C)**. The use of inhibitors of classical NK cell immune checkpoints (i.e., KIR and NKG2A) **(D)** or immune checkpoints shared with T cells (e.g., PD-1) **(E)** or, finally, a combination of these approaches represents new promising strategies in NK cell-based immunotherapy.

These findings indicate that autologous cells are not killed by NK cells thanks to an appropriate expression of all self-HLA alleles, while a wide spectrum of tumor types can be killed due to the loss of HLA molecules and to the expression/overexpression of ligands for NK cell activating receptors (Figure 1). During NK cell differentiation, CD94/NKG2A is the first HLA-I-specific receptor expressed by appearing on the most immature CD56bright NK cell subset. After several maturation steps, CD56bright cells become CD56dim, lose NKG2A, and acquire KIR receptors (26–28). The most mature NK cells are KIR+ and NKG2A– and express the marker of terminal differentiation CD57 (29).

Under normal conditions, the HLA-I-specific inhibitory receptors recognize autologous cells and prevent auto-reactive responses. However, under pathological conditions, these receptors function as ICs, by blocking the cytotoxic activity of NK cells against those tumors that maintain the expression of HLA-I molecules (11, 30).

In order to restore NK cell activity against HLA-I+ tumor cells, novel immunotherapies have been developed, based on the use of therapeutic monoclonal antibodies anti-pan-KIR2D (lirilumab) (https://www.innate-pharma.com/en/pipeline/lirilumab-first-class-anti-kir-mab-licensed-bristol-myers-squibb) and anti-NKG2A (monalizumab) (https://www.innate-pharma.com/en/pipeline/monalizumab-anti-nkg2a-mab-partnered-astrazeneca) mimicking “missing-self” response by disrupting the interaction between these ICs and their ligands. Therefore, NK cells can efficiently kill tumor cells that have lost HLA-I expression, thus becoming resistant to T lymphocytes, but also HLA-I+ cancers when blockers of ICs are used (Figure 1). These agents are currently used in phase I/II clinical trials on a range of hematologic and solid tumors as monotherapy or in combination with other agents, including other forms of IC blockade (31–37).

Notably, NK cells may also express non-HLA class I-specific inhibitory receptors such as PD-1 (38). This receptor was originally discovered on T cells and was found to exert a sharp inhibitory effect on their anti-tumor activity. In healthy donors, PD-1 is expressed on a subset of fully mature (KIR+NKG2A–CD57+) NK cells from HCMV+ individuals (38). Higher proportions of PD-1+ NK cells can be detected in patients affected by different types of tumors (36, 38, 39).

The finding that NK cells from cancer patients express PD-1 IC coupled with the observation that the use of anti-PD-1 or anti-PD-L1 monoclonal antibodies improve the anti-tumor activity of NK cells (36, 38, 39) (Figure 1) is clinically relevant for patients with tumors displaying a T-cell-resistant (HLA class Ineg) phenotype.

Recent data strongly suggest a possible role for NK cells in immunotherapeutic strategies targeting the PD-1/PD-L1 axis particularly against HLA-I-deficient tumor cells (40, 41).

NK cells also express additional constitutive or inducible IC shared with T cells, recognizing additional ligands other than HLA class I molecules. These include CTLA-4, T cell immunoglobulin- and mucin-domain-containing molecule 3 (TIM-3), lymphocyte activation gene 3 (LAG-3), T cell immunoreceptor with Ig and immunoreceptor tyrosine-based inhibition motif domains (TIGIT), and CD96 (12, 42–44).

Here, we review recent developments to improve NK cell responses against solid and hematological tumors mainly focusing on NK cell ICs.

## NK Cell-Based Therapy in Solid Tumors

Although the ability of NK cells to destroy solid tumors has been questioned, their capacity to prevent metastatic dissemination by killing circulating cancer cells is well-known. However, tumor cells frequently develop strategies to evade NK cell immunosurveillance including changes at the tumor cell level (e.g., abnormal expression of ligands for activating and inhibitory receptors) and changes in tumor microenvironment (e.g., immunosuppressive cytokines), resulting in tumor escape and cancer progression (12, 45–48). Exact mechanisms and manipulation strategies to durably and reproducibly enhance NK cell function *in vivo* are not known. However, the use of IC blockade, including lirilumab and monalizumab, to create a condition of “missing-self” recognition (consequent to the antibody-mediated disruption of pan-KIR2D or NKG2A/HLA-I interactions) may represent a promising novel therapeutic approach to cure tumor patients (49).

HLA-E is one of the emerging suppressive ligands in human tumors, and its expression negatively correlates with the overall survival (OS) of cancer patients (48). NKG2A is expressed on NK cells but also on T cells infiltrating different types of solid tumors (36, 50, 51). These findings suggested that NKG2A/HLA-E interaction could suppress the cytotoxic lymphocyte functions directly in the tumor microenvironment.

The IgG4 anti-NKG2A antibody monalizumab is currently in clinical development for the treatment of various solid tumors, either as single-agent or in combination with other compounds. In the initial clinical experiences, single-agent intravenous monalizumab was administered to patients affected by advanced gynecologic malignancies (including ovarian, endometrial, and cervical carcinomas), divided into a dose-ranging cohort and an expansion cohort, for a total of 58 patients. The drug was generally well-tolerated but achieved only short-term disease stabilization as best response (NCT02459301) (37).

In another phase II study, monalizumab was administered in combination with the anti-epithelial growth factor receptor (EGFR) antibody cetuximab in patients affected by squamous cell carcinoma of the head and neck (SCCHN) (NCT02643550). Cetuximab represents an established therapeutic approach to SCCHN acting through induction of antibody-dependent cell cytotoxicity through CD16 (FcγRIII) receptor expressed on NK cells (52). The rationale of this combination lies on the evidence that SCCHN tumors were strongly positive for HLA-E and were infiltrated with CD8+ T and NK cells, suggesting a potential sensitivity to NKG2A inhibitors. The regimen was well tolerated, being characterized mostly by grade 1–2 adverse events, and the interim analysis reported an overall response rate (ORR) of 31% and a disease stabilization rate of 54%. Although preliminary, these data appear encouraging (37).

With regard to the combination of anti-NKG2A with PD-1/PD-L1 disrupting agents, a combination of monalizumab and durvalumab has been evaluated in a first-in-human dose-escalation/dose-expansion phase I trial in patients with metastatic microsatellite-stable colorectal cancer (MSS-CRC). The rationale of this study was supported by preclinical models (https://www.innate-pharma.com/sites/default/files/180205asco_15poster_09.pdf) and was based on the hypothesis that the inhibition of NKG2A might improve the efficacy of an anti-PD-L1 antibody in a patient population characterized by poor response to PD-1/PD-L1 antibodies. The study included 40 patients in the MSS-CRC expansion cohort. The treatment was well-tolerated; in the expansion cohort, three responses and 11 disease stabilizations were observed, with a disease control rate of 24% at 16 weeks (https://ascopubs.org/doi/abs/10.1200/JCO.2018.36.15_suppl.3540). Currently, other clinical trials involving the combination of monalizumab with durvalumab in different solid tumors are ongoing (NCT03794544; NCT02671435).

The efficacy and safety of the first-in-class anti-pan-KIR2D agent lirilumab was explored in several clinical trials. In a first-in-human phase I trial, escalating doses of lirilumab were administered to patients with solid tumors (breast, kidney, or ovarian carcinoma) or hematologic malignancies. No dose-limiting toxicities were reported and maximum tolerated dose was not reached for doses up to 10 mg/kg (53). With regard to combinations including lirilumab, a phase I/II trial explored the safety of increasing doses of lirilumab in combination with the anti-PD-1 antibody nivolumab or with the anti-CTLA-4 antibody ipilimumab in a population of patients with solid tumors (136 with nivolumab; 22 with ipilimumab). Both combinations were well-tolerated, encouraging further developments. Although definitive efficacy results are not available yet, data from 29 patients with SCCHN in the lirilumab-nivolumab cohort showed an ORR equal to 24%, with durable responses. Notably, increased PD-L1 expression was strongly associated with improved probability of objective response (https://news.bms.com/press-release/bristolmyers/interim-phase-12-data-show-encouraging-clinical-benefit-lirilumab-combina). Currently, other trials designed to explore the potential role of lirilumab plus nivolumab are being conducted in populations of patients with SCCHN and invasive bladder cancer in the neoadjuvant setting (NCT03532451 and NCT03341936).

Interestingly, in those tumors resistant to anti-PD-1 immune-therapies, an up-regulation of alternative immune checkpoints, including TIM-3, has been observed. In this context, therapeutic approaches combining the administration of anti-TIM-3 and anti-PD-1 antibodies showed that the adaptive resistance to PD-1 blockade can be overcome (54).

TIM-3 is a checkpoint receptor that binds several ligands including galectin-9 (Gal-9) (55), phosphatidylserine on apoptotic cells (56), high mobility group box 1 (56), and CEA-related cell adhesion molecule-1 (57). High frequencies of circulating and/or tumor infiltrating TIM-3+ NK cells have been found in different types of malignant tumors (58–60). The increased surface levels of TIM-3 on NK cells in cancers induce NK cell impairments (61), while TIM-3 blockade results in increased NK cell cytotoxicity both *in vitro* and *ex vivo* (59, 62, 63).

TIM-3 functions as a potential prognostic marker in several tumor types, such as lung adenocarcinoma, gastric cancer, bladder cancer, and esophageal cancer (58, 59, 62, 63).

On the other hand, contradictorily, studies have also reported stimulatory functions of TIM-3 (64). These divergent functions are likely associated with the existence of multiple and different TIM-3 ligands.

An anti-TIM-3 (Sym023) antibody has been developed and is currently being tested in phase I clinical trials in patients with advanced, unresectable, and metastatic solid tumor malignancies or lymphomas that are refractory to currently available therapies, in monotherapy or in combination with anti-PD-1 or anti-LAG-3 antibodies (NCT03489343 and NCT03311412). Additional phase I studies of anti-TIM-3 antibodies have been activated in patients with advanced solid tumors, as a monotherapy or in combination with an anti-PD-1 antibody (NCT02817633, NCT03680508, NCT04139902, and NCT03744468).

LAG-3 is a negative co-inhibitory receptor expressed on T cells and NK cells that binds MHC class II (MHC-II) molecules, the C-type lectin receptor LSECtin, and a fibrinogen-like protein 1 (FGL1) on target cells (65–68). LAG-3 has been shown to suppress immune responses in several tumors, including Hodgkin's lymphoma, gastric cancer, breast cancer, and other solid tumors (69). Thus, the use of anti-LAG-3 antibodies in combination with anti-PD-1 immunotherapy has been proposed to restore T cell function (70). Although the specific role of LAG-3 on NK cells remains to be fully clarified, this inhibitory immune checkpoint is currently considered a good target for immunotherapy because of its potential to activate both T and NK cells. In this context, different anti-LAG-3 antibodies are currently being used in phase I and phase II clinical trials as single drugs in metastatic cancer, solid tumor, and lymphoma (NCT03489369 and NCT03250832) or in association with other immune checkpoints inhibitors, including anti-TIM-3 antibody, in multiple myeloma patients (NCT04150965), with anti-PD-1 antibodies in treating patients with glioblastoma (NCT02658981), solid tumors (NCT01968109), advanced malignancies including lymphoma (NCT03005782), and SCCHN (NCT04080804) or other anti-PD-1 agents in solid tumor patients (NCT02676869). A number of additional LAG-3 antibodies are currently in preclinical development.

TIGIT and CD96 are co-inhibitory receptors expressed on both T and NK cells and compete with the activating NK cell receptor DNAM-1 for binding to PVR (CD155) and Nectin-2 (CD112) (71). These receptors participate in a balanced system to control NK cell effector functions. Indeed, following interaction with their ligands, TIGIT inhibits NK cell cytotoxicity directly through its ITIM domain, whereas CD96 hampers NK cell IFN-γ production (72), thus counterbalancing DNAM-1-mediated activation. The expression of TIGIT is highly variable among different cancer types. It has been recently demonstrated that TIGIT is highly expressed on tumor-infiltrating NK cells and associated with NK cell exhaustion in different tumor models and patients with colon cancer (73). Notably, the therapeutic effects of anti-TIGIT, anti-PD-L1, or anti-TIGIT antibodies combined with anti-PD-L1 antibodies depended on the presence of NK cells (73), indicating the importance of NK cells in checkpoint-targeted immunotherapy. Currently, several ongoing clinical trials (phase I and phase II) focus on testing the feasibility of targeting TIGIT pathway and improving therapeutic effects through combination with existing immunotherapies, including anti-PD-1 agents (NCT04150965, NCT03119428, NCT04047862, and NCT03563716), mainly in solid tumor patients.

Recently, results from clinical studies have demonstrated the safety of the infusion of allogeneic NK cells for immunotherapy of both hematological malignancies and solid tumors (74) despite the microenvironment rich of NK-inhibiting factors and the limited ability of infiltration of immune cells. While generally safe, allogenic NK cell infusions generally showed poor anti-tumor activity.

Therefore, strategies designed to improve the efficacy of adoptive transfer of NK cells are currently being explored and include associations with IC inhibitors or chemotherapy, induction of chemokine expression that can improve NK cell migration and trafficking into tumors, as well as concurrent administration of cytokines with activating effect on NK cells. However, while some of such approaches achieved encouraging results, none of these has evolved into an established protocol; hence, additional efforts in this setting are warranted (75–79). Relevant trials are reported in Table 1.

**Table 1:** Relevant clinical trials involving immune checkpoint blockade in NK cells.

**Trial**	**Targets**	**Setting**	**Agent**	**Phase**	**Pts**	**Results**
**SOLID TUMORS**						
Tinker et al. (37)	NKG2A	Advanced gynecological solid tumors	Monalizumab	I	58	Manageable safety profile; short term response
NCT02643550	NKG2A; EGFR	Advanced squamous cell carcinoma of the head and neck	Monalizumab plus cetuximab	I/II	31	Manageable safety profile; ORR: 31%; DCR: 54%
Segal et al. (80)	NKG2A; PD-L1	Metastatic microsatellite-stable colorectal cancer	Durvalumab plus monalizumab	I	40	Ongoing; preliminary data show manageable safety profile and DCR: 24% at 16 weeks
NCT03794544	NKG2A; PD-L1	Resectable non-small cell lung cancer	Durvalumab plus monalizumab	II	160 (estimated)	Ongoing
Vey et al. (53)	KIR2D	Solid and hematologic malignancies	Lirilumab	I	37	No reported dose-limiting toxicity
NCT03203876	KIR2D; PD-1; CTLA-4	Solid tumors	Lirilumab plus nivolumab with or without ipilimumab	I/II	21 (estimated)	Ongoing
NCT03532451	KIR2D; PD-1	Resectable bladder cancer	Nivolumab with or without lirilumab	I	43 (estimated)	Ongoing
NCT03341936	KIR2D; PD-1	Resectable squamous cell carcinoma of the head and neck	Nivolumab plus lirilumab	II	58 (estimated)	Ongoing
NCT03489343	TIM-3	Advanced solid tumors or lymphomas	Sym023	I	48 (estimated)	Ongoing
NCT03311412	TIM-3; LAG-3	Advanced solid tumors or lymphomas	Sym021 with or without Sym022 or Sym023	I	102 (estimated)	Ongoing
NCT02817633	TIM-3	Advanced solid tumors	TSR-022	I	873 (estimated)	Ongoing
NCT03680508	TIM-3; PD-1	Liver cancer	TSR-022 plus TSR-042	II	42 (estimated)	Ongoing
NCT04139902	TIM-3; PD-1	Resectable melanoma	TSR-042 with or without TSR-022	II	56 (estimated)	Ongoing
NCT03744468	TIM-3; PD-1	Solid tumors	BGB-A425 plus tislelizumab	I/II	162 (estimated)	Ongoing
NCT03489369	LAG-3	Advanced solid tumors or lymphomas	Sym022	I	30 (estimated)	Ongoing
NCT03250832	LAG-3	Advanced solid tumors	TSR-033 alone or in combination with PD-1 blocking agents	I	200 (estimated)	Ongoing
NCT04150965	LAG-3; TIGIT	Multiple myeloma	Elotuzumab	I/II	104 (estimated)	Ongoing
NCT02658981	LAG-3; PD-1	Recurrent glioblastoma	BMS-986016 with or without nivolumab	I	100 (estimated)	Ongoing
NCT01968109	LAG-3; PD-1	Advanced solid tumors	BMS-986016 with or without nivolumab	I/II	2,000 (estimated)	Ongoing
NCT03005782	LAG-3; PD-1	Advanced solid tumors	REGN3767 with or without REGN2810	I	589 (estimated)	Ongoing
NCT04080804	PD-1; LAG-3; CTLA4	Advanced head and neck squamous cell carcinoma	Nivolumab with or without BMS-986016 or ipilimumab	II	60 (estimated)	Ongoing
NCT02676869	LAG-3; PD-1	Advanced melanoma	IMP321 plus pembrolizumab	I	24 (estimated)	Ongoing
NCT03119428	TIGIT; PD-1	Advanced solid tumors	OMP-313M32 with or without nivolumab	I	33 (estimated)	Ongoing
NCT04047862	TIGIT; PD-1	Advanced solid tumors	BGB-A1217 plus tislelizumab	I	39 (estimated)	Ongoing
NCT03563716	TIGIT; PD-L1	Advanced non-small cell lung cancer	MTIG7192A plus atezolizumab	II	135 (estimated)	Ongoing
**HEMATOLOGICAL MALIGNANCIES**						
Vey et al. (33)	KIR2D	Acute myeloid leukemia	IPH2101	I	23	Manageable safety profile
Korde et al. (81)	KIR2D	Smoldering multiple myeloma	IPH2101	II	9	Failure to meet the primary endpoint (50% decline in M-protein)
NCT01592370	KIR2D; PD-1	Multiple myeloma	Lirilumab plus nivolumab (among multiple arms including nivolumab)	I/II	375 (estimated; multiple arms)	Ongoing
Guolo et al. (82)	PD-1	Relapsed or refractory Hodgkin lymphoma	Nivolumab supported by the reinfusion of unselected autologous lymphocytes	I/II	7	Manageable safety profile; fast immune recovery
NCT02557516	KIR2D	Chronic lymphocytic leukemia	Monalizumab plus ibrutinib	I/II	22 (estimated)	Ongoing
NCT03066648	PD-1; TIM-3	Acute myeloid leukemia; high-risk myelodysplastic syndrome	PDR001 and/or MBG453 in combination with Decitabine	I	235 (estimated)	Ongoing

*References and additional details can be found in the text. For published trials, we included the name of the first author, while for ongoing trials, we included NCT identifier. The trials are reported in the same order of the main text. Pts, patients*.

## NK Cells to Treat Hematological Malignancies

Current approaches of NK cell immunotherapy for hematological malignancies involve methods for *in vivo* potentiation of NK cell proliferation and activity; adoptive transfer of NK cells from autologous and allogeneic sources, and NK cell lines; genetic modification of NK cells; and, similar to solid tumor, the use of IC blockade (12, 83).

Following the introduction of haploidentical hematopoietic stem cell transplantation (haplo-HSCT), the potential anti-leukemic activity of donor-derived alloreactive NK cells has been observed (84–87). After engraftment, NK cells are the first lymphocyte subset that appears in peripheral blood, suggesting their role as graft-vs.-leukemia effector cells, in the absence of GvHD (84, 88–90). The generation of alloreactive NK cells (i.e., NK cells expressing inhibitory KIRs that are not engaged by any of the HLA-I alleles present on allogeneic target cells) results in improved clinical outcome in haplo-HSCT thanks to their ability to kill not only leukemic blasts (GvL) but also patient dendritic cells (DCs) (91) and T cells, thus preventing GvHD and HvG reaction, respectively (92–95).

Given the crucial role of alloreactive NK cells in mediating safe and durable anti-tumor immunity in patients receiving KIR/HLA-C-mismatched transplantation, different strategies, including miRNA targeting the expression of HLA-C-specific KIRs (i.e., KIR2DL1, KIR2DL2/L3) (96) or therapeutic antibody blocking these inhibitory KIRs (lirilumab) for HLA-C, were generated to simulate the mechanism of “missing self” condition.

The IgG4 antibody lirilumab prevents inhibitory signals from pan-KIR2D, increasing NK cell-mediated tumor killing of AML *in vitro* and *in vivo* (31). This anti-pan-KIR2D antibody had acceptable safety without significant toxicity or autoimmunity in AML and multiple myeloma (MM) patients (33, 97). Although lirilumab failed to show significant efficacy as a monotherapy in MM, dual immune therapy with lenalidomide showed a good response on relapsed/refractory (R/R) MM in clinical trials (81, 97). Lirilumab is currently being widely tested in combination with other therapeutics, including rituximab (an anti-CD20 antibody), and other forms of IC blockade, such as nivolumab in R/R non-Hodgkin's lymphoma, Hodgkin's lymphoma (HL), and MM patients (NCT01592370) (34, 97).

In R/R HL patients (98–102), IC inhibitors have shown good activity; indeed, nivolumab is currently approved for this indication by the Food and Drug Administration and the European Medicines Agency (103). Interestingly, the Hodgkin neoplastic cells (Reed Sternberg cells) hyper-express PD-L1 but have low/negative expression of HLA-I molecules. This means that NK cells that exert their killing activity mainly against HLA-Ineg targets, but not cytotoxic T CD8+ cells, may be the primary effectors in the immune response induced by nivolumab in HL patients. In this view, an increase in cytotoxic NK cell population during IC blockade treatment in HL patients has been recently observed (104). In order to better elucidate the exact mechanism of action of nivolumab in HL and to improve the efficacy in terms of complete response (CR) in R/R HL, an innovative clinical protocol based on the combined application of high-dose chemotherapy with autologous stem cell transplant (ASCT) and early post-transplant administration of nivolumab, supported by autologous lymphocytes re-infusions (ALI), has been recently proposed (https://doi.org/10.1182/blood-2018-99-118901). Preliminary observations support the hypothesis that NK cells play a primary role in response to nivolumab in HL patients (82, 104). Furthermore, from a clinical point of view, preliminary data are encouraging, as all six R/R patients treated so far achieved CR (82).

Leukemic cells have been shown to overexpress HLA-E, a ligand for NKG2A. This provides the rationale for the use of monalizumab for the treatment of leukemia. The use of monalizumab in HSCT has recently also been proposed to induce NK cell alloreactivity in the first weeks after transplant when almost all reconstituting NK cells are NKG2Apos, in order to limit opportunistic infection and leukemia relapse (105). Moreover, monalizumab is currently under evaluation in several phase I/II clinical trials in monotherapy (35) or in combination with other therapeutic antibodies, such as the Bruton's tyrosine kinase inhibitor ibrutinib, in patients with R/R or previously untreated chronic lymphocytic leukemia (NCT02557516).

A phase I clinical trial evaluating the hypomethylating agent decitabine together with either PDR001 (anti-PD-1 antibody), MBG453 (anti-TIM-3 antibody), or their combination is currently recruiting patients with R/R AML patients not eligible for intensive therapy, as well as high-risk myelodysplastic patients (NCT03066648).

Another anti-TIM-3 antibody (Sym023) is currently being tested in phase I clinical trials in patients with lymphoma refractory to currently available therapies, in monotherapy or in combination with anti-PD-1 (Sym021) or anti-LAG-3 (Sym022) antibodies (NCT03489343 and NCT03311412).

The anti-LAG-3 antibody BMS-986016 is currently being used in phase I and phase II clinical trials in combination with nivolumab in subjects with relapsed or refractory HL, and relapsed or refractory diffuse large B cell lymphoma (DLBCL) (NCT02061761) or as a single drug in lymphoma (NCT03489369). Relevant trials are reported in Table 1.

## Concluding Remarks

Several molecular mechanisms regulating the anti-tumor activity of NK cells have been discovered over the last decades. However, further characterization of the main immunosuppressive pathways developed by tumor cells to evade NK cell recognition is still needed. Analysis of NK cells and tumor cells and their relationship is necessary to define personalized immunotherapy procedures in cancer patients.

The combined blockade of checkpoint molecules expressed by T cells and NK cells could trigger antitumor immunity mediated by innate and adaptive populations, allowing the two approaches to complement each other. NK cell-targeted immunotherapy may provide an alternative, or a complementary approach, to overcome the limitations of T-cell immunotherapy. In addition, combination with NK cell immunotherapy could increase the response rate of treatments targeting T cells (Figure 1 and Table 1).

In conclusion, considering the excellent outcome of some patients, future efforts should be addressed to identify the best inhibitory pathways to target for future clinical applications. Moreover, further studies should aim at improving NK cell-based immunotherapy by targeting the tumor-induced NK cell inhibition, thus promoting the maximal anti-tumor effect of these innate effectors.

## Author Contributions

All authors listed have made a substantial, direct and intellectual contribution to the work, and approved it for publication.

### Conflict of Interest

The authors declare that the research was conducted in the absence of any commercial or financial relationships that could be construed as a potential conflict of interest.
